# Perceived life balance among young adult students: a comparison between caregivers and non-caregivers

**DOI:** 10.1186/s40359-023-01500-z

**Published:** 2024-01-08

**Authors:** Srishti Dang, Anne Looijmans, Giovanni Lamura, Mariët Hagedoorn

**Affiliations:** 1 Department of Health Psychology, University of Groningen, University Medical Center Groningen, Groningen, The Netherlands; 2 INRCA IRCCS - National Institute of Health and Science On Aging, Ancona, Italy

**Keywords:** Informal caregivers, Life balance, Young adults, Psychological functions, Students

## Abstract

**Background:**

Young adult caregivers (YACs) are individuals aged 18–25 years who provide care to a loved one (parent, sibling) with frailty, disability, or illness. As young adults, the transition period between adolescence and adulthood can be more challenging for YACs than their peers without care responsibilities (non-YACs), as they have to integrate caregiving with other life areas (education, relationships). This study compared the perceived life balance and the psychological functioning (i.e., burnout, negative and positive affect, and life satisfaction) between YACs and non-YACs.

**Method:**

An online cross-sectional survey was conducted among 74 YACs (85.1% females, 22.0 ± 2.1 years) and 246 non-YACs (76.0% females, 21.8 ± 2.0 years) studying in the Netherlands. The survey assessed demographic characteristics, caregiving characteristics (to be filled out only by the YACs), life balance, and psychological functioning. We used Chi-square tests for categorical variables and independent T-tests for continuous variables to examine possible differences in demographic characteristics between YACs and non-YACs. In addition, we used independent T-tests to compare the perceived life balance and psychological functioning between YACs and non-YACs.

**Results:**

YACs and non-YACs were similar on all the demographic characteristics, except for living status; fewer YACs (44.6%) than non-YACs (59.3%) lived on their own, with or without other students/friends (*χ*^*2*^ = *16.3, p* = *0.01*). YACs perceived slightly less balance in life than non-YACs *(d* = *-.29, p* = *.03*). Both groups did not differ in experiencing burnout, affect, and life satisfaction (all *p* > *.05*). They experienced high levels of burnout and moderate levels of life satisfaction.

**Discussion:**

Although YACs perceived a little less balance in life than non-YACs, this was not reflected in their psychological functioning. Healthcare professionals and school counselors may need to recognise the critical phase of all young adults and provide the support that could, for example, help them reduce burnout and enhance their quality of life.

## Background

Our social and health care systems rely to a large extent on individuals who provide unpaid assistance or care to a person with frailty, a chronic illness, or a disability [48]. These individuals are called informal caregivers (ICGs), and they cover an estimated 70 to 95 percent of all the care provided [55]. They assist the care recipient with activities such as bathing, cooking, and managing finances [48, 52]. Caregiving may impact different areas in ICG’s lives, such as employment (i.e., part-time or full-time job), social life (i.e., friends, family), personal time (i.e., leisure time, pursuing hobbies), and, in case of young adult caregivers (YACs), education [8, 22, 23, 31]. It is estimated that approximately 25% of the young adults (aged 16–24 years) in the Netherlands are taking care of a family member [47]. In the literature, YACs are most often defined as ICGs in the age of 18–25 years, who make up 12 to 18% of the total population of ICGs, but they are often ignored as caregivers by the society [34].

It is important to recognise YACs as caregivers because they are in the critical transition period between adolescence and adulthood. In this phase, they explore the possibilities in life open to them and make enduring choices in different life areas, such as education, career, and relationships [5, 45, 54]. This phase can be more challenging for YACs than their peers, as they have to integrate caregiving responsibilities with the activities in other areas of their life [10, 17, 27, 28].

Overall, the literature suggests that YACs do face difficulties in finding ways to fulfill personal, social, and professional goals because of their care responsibilities [9, 28, 46]. Qualitative studies exploring the challenges that YACs face in the pursuit of education suggest that caring responsibilities limit their attendance and academic participation at college [16, 27, 32, 36, 42, 46]. YACs find it challenging to maintain study routines, keep up with coursework, and devote sufficient quality time and effort to their homework while providing care. They feel less satisfied with their academic performances and achievements than their peers [16]. In line with these qualitative studies, a quantitative study showed that YACs scored lower grades in college than young adults without caregiving responsibilities (non-YACs) [36]. In addition, YACs have indicated to experience a loss of personal time due to caregiving responsibilities. As a result, they have fewer opportunities to pursue their leisure activities, connect with their peers in college, or maintain their relationships with their friends and close ones [26, 27]. These findings indicate that YACs face challenges in different life areas due to caregiving, which may lead to a lower life balance [42].

Life balance as defined by Gröpel [26] is the degree to which one is able to spend appropriate time on each of the most important life areas, that is, social life, employment, health, and meaningfulness of life [26]. Previous research has mostly explored balance in life with respect to employment and caregiving among ICGs in general through qualitative studies, concluding that ICGs find it challenging to balance caregiving with employment [13, 42, 56]. To the best of our knowledge, there are no studies explicitly focusing on perceived life balance among YACs. Considering that YACs are in the phase of exploring different life areas while juggling with caregiving responsibilities, it becomes important to gain more insight into how they perceive their life balance, as a lower life balance may have a negative impact on their wellbeing.

Lower life balance is assumed to be associated with lower psychological functioning [1, 24, 29, 38]. In line with this, a previous study among university students has indicated a positive correlation between life balance and positive outcomes (such as life satisfaction), and a negative association with anxiety and depression [26]. Although studies that link life balance to psychological functioning in YACs are lacking, we do know that demanding care responsibilities at an early stage of life can be burdensome and contribute to lower psychological functioning, as reflected in symptoms of depression, anxiety, and insomnia [7, 9, 28]. Quantitative studies comparing YACs and non-YACs suggest that YACs experience significantly higher levels of depression and anxiety than non-YACs [7, 25]. Interestingly, no difference was found between the two groups on life satisfaction [36]. However, one study among YACs suggested that an increase in the number of hours of care was associated with a decrease in life satisfaction [29].

In light of the findings highlighted above, the primary aim of this study is to compare perceived life balance and satisfaction with time spent on activities in different life areas (i.e., social life, employment, education, and personal life) in YACs and non-YACs. As a secondary aim, we will compare burnout, negative and positive affect, and life satisfaction between YACs and non-YACs, and look into the relationship between perceived life balance and psychological functioning. We hypothesise that YACs, compared to non-YACs, will perceive less balance in life, are less satisfied with the time spent in different life areas, report higher levels of burnout and negative affect, and lower levels of positive affect and life satisfaction. We hypothesise that perceived life balance will be negatively related to burnout and negative affect and positively related to life satisfaction and positive affect in both YACs and non-YACs.

## Method

### Study design

A cross-sectional survey study was conducted to compare the perceived life balance and psychological functioning (burnout, negative and positive affect, and life satisfaction) between YACs and non-YACs. The survey was administered online using Qualtrics platform. The Central Ethics Review Board of University Medical Center Groningen, The Netherlands approved the study with the registration number 202000623.

### Participants and recruitment

The study sample represents student YACs and non-YACs in the Netherlands. Participants were eligible to participate in the study if they were between 18–25 years of age and were a student at the university, university of applied sciences, or secondary vocational education in the Netherlands. Participants were recruited by (i) posting the survey on social networks such as Facebook groups, LinkedIn, and Twitter; (ii) contacting educational institutions to disseminate the survey among students; and (iii) contacting caregiving organisations in the Netherlands to reach the YACs group.

### Data collection

Data collection started in December 2020 and finished in March 2022. The survey could be completed in either Dutch or English. Participants completed a question about their age and student status to determine their eligibility to participate in the study. When eligible, participants were asked to read the study information on the study’s objectives, procedures, risks, benefits, and their right to withdraw from the study any time they want. Participants who provided digital informed consent continued to fill out the survey, which took approximately 10–15 min to complete. Data collected during the study were stored on the secure server of the UMCG.

### Measures

The survey consisted of questions on demographic characteristics, caregiving characteristics (to be filled out only by the YACs), and a collection of validated scales (i.e., life balance, burnout, negative and positive affect, and life satisfaction) to measure the relevant variables described below.

#### Demographic characteristics

Participants completed questions about their age, gender, education, nationality, relationship status, employment status, living situation (i.e., whom do they live with), and financial troubles in general or because of COVID-19.

#### Caregiving status and characteristics

To determine whether participants were YACs or non-YACs, the following question was asked: *‘Do you take care of a loved one (e.g., parent, grandparent, sibling, spouse, partner, relative, friend, or neighbor) who is living with a disability, chronic illness, mental illness, old age problems, or substance abuse?’.* Participants responding *‘No’* were categorised as non-YACs and participants responding *‘Yes, I am the one who provides maximum care to my loved one’* or *‘Yes, I occasionally provide care to my loved one’* were categorised as YACs. Participants in the YACs group were asked to indicate who they cared for, illness of the care recipient, if there is any other person providing care to their loved one, hours per week spent on caregiving, caregiving tasks they perform, and if their loved one was diagnosed with COVID-19.

#### Perceived life balance

We assessed two variables, namely overall perceived life balance and satisfaction with time spent on different life areas (social life, education, caregiving, and personal life). Overall life balance was measured with the occupational balance questionnaire (OBQ). This questionnaire measures the perceptions of meaningful occupation and the need for more meaningful occupation [58]. The scale has been adapted for this study by replacing the word ‘occupations' with the term 'activities.' Although ‘occupations' is a synonym for ‘activities’ in this scale, which represents all the activities in different life areas, it may have been confused with ‘job’ or ‘work’. The scale consists of 13-items measured on a six-step ordinal scale ranging from 0 (completely disagree) to 5 (completely agree). The scores of all items were summed up, ranging from 0 to 65, where a higher score indicates a higher overall life balance. The occupational balance questionnaire has good internal consistency (Cronbach's alpha, 0.94) and sufficient test–retest reliability (Spearman's Rho, 0.93) [35]. The scale also shows good reliability in the current sample of YACs and non-YACs, with a Cronbach’s alpha of 0.88.

In addition, satisfaction with time spent in particular life areas (i.e., social life, employment, education, personal life and, caregiving) was assessed. For example, *‘I am satisfied with the time I spend on my work’*. The satisfaction with time spent in employment and caregiving were only filled out by participants who were employed and caregivers, respectively. The questions were assessed on a Likert scale ranging from 0 (completely disagree) to 5 (completely agree). A high score in a question focusing on a particular life area indicates high satisfaction with time spent in that area.

#### Hours spent on different activities

We asked the participants to fill in the number of hours spent in different life areas, i.e., social life, employment, education, personal time and, caregiving. The life areas, employment, and caregiving were only filled out by participants who were employed and caregivers, respectively.

#### Burnout

Burnout was measured using the general Burnout Assessment Tool (BAT) [50]. The 22-item BAT scale measures the core symptoms of burnout through four core sub-scales: exhaustion, mental distance, emotional impairment, and cognitive impairment. For our study, we assessed the three core sub-scales: exhaustion, eight items (example: ‘*When I get up in the morning, I lack the energy to start a new day*’), emotional impairment, five items (example ‘*I may overreact unintentionally*’) and, cognitive impairment, five items (example: ‘*I struggle to think clearly*’). We did not include the sub-scale mental distance, since this sub-scale was focused on work, and we aimed to use the general version of the BAT to examine the overall levels of burnout.

The items were assessed on a five-point Likert scale ranging from 1 (never) to 5 (always). We calculated the mean score for each sub-scale. Higher mean scores represent higher levels of the burnout symptoms. The mean score for the burnout sub-scales, exhaustion, emotional impairment and cognitive impairment are interpreted as low, average, high or very high based on certain cut-off values [50]. Cronbach’s alpha ranged from 0.90 to 0.92 for the sub-scales and was 0.95 for the total core BAT scale [50]. In the current sample, the three core sub-scales showed good reliability, with a Cronbach's alpha of 0.90 for exhaustion, 0.83 for emotional impairment, and 0.89 for cognitive impairment.

#### Negative and positive affect

Affect was measured using the Positive and Negative Affect Schedule (PANAS) [59]. The PANAS contains two sub-scales, positive affect (PA) and negative affect (NA), of 10-items each. The items are answered on a five-point scale, ranging from 1 (very slightly or not at all) to 5 (extremely). PA reflects the extent to which a person feels enthusiastic, active, and alert. NA reflects the extent to which a person feels distressed, indicating aversive mood states. A sum score is obtained separately for PA and NA by adding up the scores for the respective items. The internal consistency (Cronbach's alpha) of the PA and NA scale are all acceptably high, ranging from 0.86 to 0.90 for PA and from 0.84 to 0.87 for NA [59]. The scale also shows good reliability in the current sample, with a Cronbach’s alpha of 0.82 for PA and 0.85 for NA.

#### Life satisfaction

Life satisfaction was measured using the five-item Satisfaction with Life Scale (SWLS) [41]. This scale measures life satisfaction as a whole and not within a specific life area. The items are presented on a seven-point scale, ranging from 1 (strongly disagree) to 7 (strongly agree). A sum score is calculated and can range from 5 to 35, with a score between 31–35 classified as extremely satisfied, 26–30 as satisfied, 21–24 as slightly satisfied, 20 as neutral, 15–19 as slightly dissatisfied, 10–14 as dissatisfied, and 5–9 as extremely dissatisfied [41]. The SWLS has good internal consistency (Cronbach's alpha, 0.83) [40]. The scale also shows good reliability in the current sample, with a Cronbach’s alpha of 0.78.

Two additional questions were added at the end of the survey to examine the impact of COVID-19 on life balance (*‘I perceive less balance among different activities in the times of COVID-19 as compared to before’*) and mental well-being (*‘I experience reduced mental well-being in the times of COVID-19 as compared to before’*) of the participants. The question on impact of COVID-19 on life balance was measured on a six-step ordinal scale ranging from 0 (completely disagree) to 5 (completely agree) response options. The question on impact of COVID-19 on mental well-being was measured on a dichotomous scale having ‘Yes’ and ‘No’ response option. Percentage of participants were calculated for each response option for both the questions.

### Data analysis

IBM SPSS version 28 was used for complete analysis. We first described the demographic characteristics for YACs and non-YACs, and caregiving characteristics for YACs using frequencies and percentages as appropriate. We examined possible differences in demographic characteristics between the two groups using Chi-square tests (categorical variable) and independent T-tests (continuous variable). We then checked the normality of the continuous outcome variables graphically (normal q-q plots and histogram) and by using Kolmogov-Smirnov and Shapiro–Wilk tests.

To compare the perceived life balance and psychological functioning (burnout, negative and positive affect, and life satisfaction) between YACs and non-YACs, we used independent T-tests. The effect sizes of the mean differences were calculated using Cohen’s d. An effect size of 0.2 is considered small, 0.5 as medium, and an effect size greater than or equal to 0.8 as large [51]. The relationship between perceived life balance and psychological functioning was determined using bivariate correlation analysis. In all analyses, the level of statistical significance was set at 0.05 (two-tailed).

## Results

### Participants and demographic characteristics

Of the total 354 participants who started filling out the survey, we included participants in our analysis who completed the primary outcome, that is, the overall perceived life balance (*N* = 320, 90.4%). Of these 320 participants, 74 were YACs, and 246 were non-YACs. Table 1 shows that the YACs and non-YACs were similar in all the demographic characteristics, except for living status: fewer YACs (44.6%) than non-YACs (59.3%) lived on their own, with or without other students/friends (χ^2^ = 16.3, *p* = 0.01). Participants in both groups were predominantly female (YACs: 85.1%, non-YACs: 76.0%) and of Dutch nationality (YACs: 79.7%, non-YACs: 70.3%). With respect to the relationship status, more YACs (63.5%) than non-YACs (48.8%) were single, although the difference was not significant.

**Table 1:** Demographic characteristics of student YACs and non-YACs (Total *N *= 320)

*Demographic characteristics*	*YACs (N* = *74)*	*Non-YACs (N* = *246)*	χ^2^ / t	*p*
**Age (range 18–25)**, **years, M ± SD**	22.0** ± **2.1	21.8** ± **2.0	0.8	.66
**Gender**				
Female	63 (85.1)	187 (76.0)	3.7	.30
Male	10 (13.5)	54 (22.0)		
Other	1 (1.4)	5 (2.0)		
**Education level, N (%)**				
University	32 (43.2)	125 (50.8)	1.7	.64
Applied university	39 (52.7)	110 (44.7)		
Secondary vocational education	3 (4.1)	11 (4.5)		
**Nationality, N (%)**				
Dutch (From Netherlands)	59 (79.7)	173 (70.3)	2.5	.11
Others (Migrated to the Netherlands)	15 (2.3)	73 (29.7)		
**Relationship status, N (%)**				
Single	47 (63.5)	120 (48.8)	5.4	.14
In a relationship	26 (35.1)	121 (49.2)		
Married/partner	1 (1.4)	3 (1.2)		
Others	n/a	2 (0.8)		
**Living status, N (%)**				
With parents	8 (10.8)	26 (10.6)	16.3	.01*
With parents and sibling(s)	20 (27.0)	40 (16.3)		
With family members like grandparent(s), uncle, aunt	1 (1.4)	2 (0.8)		
On my own, with other students/friends	20 (27.0)	93 (37.8)		
On my own, without anyone	13 (17.6)	53 (21.5)		
With my partner/spouse	6 (8.1)	29 (11.8)		
Others	6 (8.1)	3 (1.2)		
**Employment status (having a (side) job), N (%)**				
Yes	48 (64.9)	149 (60.6)	0.4	.51
No	26 (35.1)	97 (39.4)		
**Experiencing financial trouble, N (%)**				
Yes	19 (25.7)	53 (21.5)	0.6	.46
No	55 (74.3)	193 (78.5)		
**Of those experiencing financial trouble, this is due to COVID-19, N (%)**				
Yes	9 (47.4)	23 (43.4)	0.09	.77
No	10 (52.6)	30 (56.6)		
**Diagnosed with COVID-19, N (%)**				
Yes, admitted to the hospital	1 (1.4)	1 (0.4)	7.9	.10
Yes, not admitted to the hospital	8 (10.8)	20 (8.1)		
No, but might have had it	14 (18.9)	27 (11.0)		
No	51 (68.9)	198 (80.5)		

*N* = *Total number of participants; M* = *Mean; SD* = *Standard deviation;* χ^2^ values for categorical variables; t value for continuous variable (age); *Results are significant at a p-value of 0.05

### Caregiving characteristics

Table 2 displays the caregiving characteristics of YACs, showing that most YACs provided care to a parent (33.8%) followed by a grandparent (27.0%) and sibling (17.6%). They mostly provided care to a care recipient with a mental illness (31.1%) followed by a chronic illness (24.3%), such as heart disease and cancer. Although participants varied in the duration of care provided to their care recipient, almost one out of two YACs (48.5%) were providing care for more than five years. Moreover, YACs spent, on average, 8.6 h per week on caregiving (see Table 3), and they were most often responsible for providing emotional support (83.8%) and doing household tasks (73.0%). Most of the YACs were not living with the care recipient (66.2%) and were sharing caregiving responsibility with other family members or friends (85.1%).

**Table 2:** Caregiving characteristics of student YACs (Total *N *= 74)

Caregiving characteristics	*YACs*
**Caregiving status, N (%)**	
Provides maximum care to the care recipient	12 (16.2)
Occasionally provide care to the care recipient	62 (83.8)
**ICGs provide care to a, N (%)**	
Parent	25 (33.8)
Grandparent	20 (27.0)
Sibling	13 (17.6)
Spouse/partner	5 (6.8)
Friend	5 (6.8)
Other	6 (8.1)
**Illness of the care recipient, N (%)**	
Mental Illness	26 (35.1)
Chronic Illness	18 (24.3)
Fragility	13 (17.6)
Disability	6 (8.1)
Substance abuse	1 (1.3)
Co-morbidity	5 (6.8)
Other	5 (6.8)
**Living with the care recipient, N (%)**	
Yes	25 (33.8)
No	49 (66.2)
**Anyone else providing care, N (%)**	
Yes	63 (85.1)
No	11 (14.9)
**Duration of care (in years), N (%)**	
< 1	6 (8.1)
1–3	30 (40.5)
4–6	13 (17.6)
7–9	13 (17.6)
> = 10	12 (16.2)
**Caregiving responsibilities, N (%)**	
Emotional support	62 (83.8)
Household tasks	54 (73.0)
Practical support	29 (39.2)
Personal care	16 (21.6)
Other	4 (5.4)
**Care recipient diagnosed with COVID-19, N (%)**	
Yes, admitted to the hospital	1 (1.4)
Yes, not admitted to the hospital	6 (8.1)
No, but might have had it	6 (8.1)
No	61 (82.4)

**Table 3:** Perceived life balance, hours spent in different life areas and psychological functioning scores for YACs and non-YACs

*Variables*	*YACs*			*Non-YACs*			*t*	*p*	*Cohen’s d*
	***N***	***M***	***SD***	***N***	***M***	***SD***			
**Perceived life balance**									
Overall life balance	74	34.8	9.8	246	37.2	8.1	-2.15	.03*	-.29
Satisfaction of time spent in particular life areas									
Social life	61	3.9	0.6	204	3.4	0.6	2.76	.001*	.39
Employment	38	4.0	0.9	116	4.0	1.0	-.08	1.00	-.11
Education	61	3.7	1.0	204	3.8	0.9	-.59	.66	-.06
Personal time	61	3.5	1.0	204	3.7	0.9	-1.32	.20	-.19
Caregiving	58	3.9	0.8	n/a	n/a	n/a	n/a	n/a	n/a
**Hours spent on different life areas (hours/week)**									
Social life	67	9.6	12.4	237	10.5	8.3	-.75	.46	-.10
Employment	43	12.8	9.7	144	10.9	7.1	1.43	.16	.20
Education	67	29.0	13.1	237	29.8	14.0	-.44	.66	-.06
Personal time	67	11.5	11.5	237	15.0	11.6	-2.19	.03*	-.30
Caregiving	64	8.6	8.2	n/a	n/a	n/a	n/a	n/a	n/a
**Psychological functioning**									
Burnout									
Exhaustion	65	3.2	0.9	228	3.1	0.8	1.57	.15	.22
Emotional impairment	65	3.0	0.8	228	2.9	0.9	.91	.36	.13
Cognitive impairment	65	3.1	1.0	228	3.0	0.8	1.19	.28	.17
Negative and positive affect									
Negative affect	65	23.3	7.3	230	21.6	7.1	1.76	.09	.23
Positive affect	65	31.0	6.5	230	31.1	6.6	-.20	.84	-.03
Life satisfaction	61	21.6	6.5	218	22.1	5.8	**-.62**	.54	-.09

*N* Number of participants*, M* Mean*, SD* Standard deviation*, d* *Cohen’s d; p* = *significant value (p* < *0.05)*

^*^Results are significant at a p-value of 0.05

### Perceived life balance

#### Hypothesis 1: YACs perceive less balance in life than non-YACs

The results show that YACs perceived less balance in life (*M* = 34.8, *SD* = 9.8) than non-YACs (*M* = 37.2, *SD* = 8.1; *p* = 0.03, see Table 3). This difference was considered small based on the effect size of -0.29.

#### Hypothesis 2: YACs are less satisfied with the time spent on different life areas (social life, employment, education, personal time) than non-YACs

The results show that, except for social life, there were no significant differences between YACs and non-YACs in their satisfaction with time spent in the life areas; employment, education, and personal time. For social life, YACs (*M* = 3.9, *SD* = 0.6) were significantly more satisfied with the time spent on social life than non-YACs (*M* = 3.4, *SD* = 0.6; *p* = 0.001, see Table 3). For other life areas (employment, education, and personal time), both the groups recorded a mean score above 3.4 out of 6.0 for each area of life, which might indicate that both groups were rather satisfied with the time they spent on different activities.

**Fig. 1:**
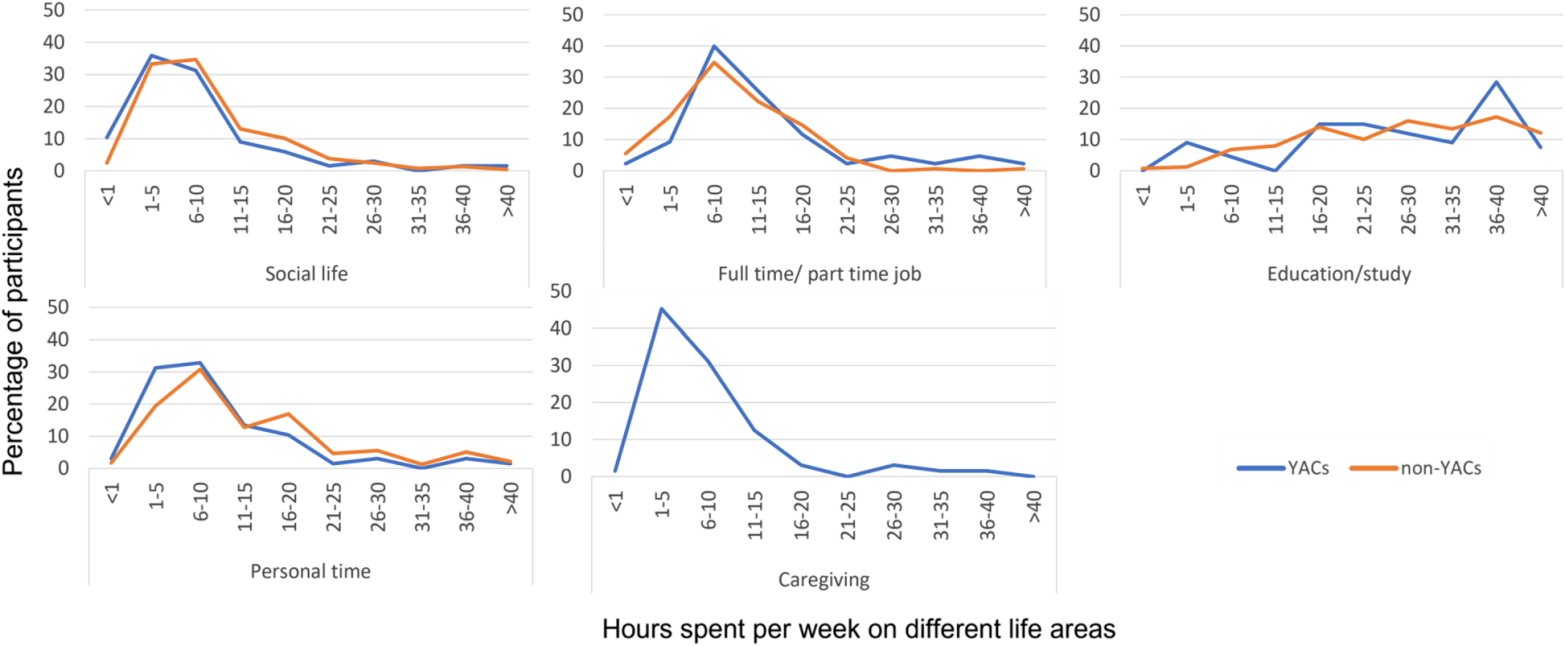
Hours spent per week on different activities (education, job, social life, personal time, and caregiving) by YACs and non-YACs

Moreover, if we look at the estimated hours spent on each life domain, YACs and non-YACs did not significantly differ in the hours spent on their social life, employment, and education (see Table 3 and Fig. 1). Both groups spent most of their time on education (YACs = 29.0 h/week; non-YACs = 29.8 h/week). However, YACs spent, on average, 3.5 h per week less on personal time (*M* = 11.5, *SD* = 11.5) than non-YACs (*M* = 15.0, *SD* = 11.6; *p* = 0.03). Based on the effect size of -0.30, this difference was considered small.

#### Hypothesis 3: YACs report higher levels of burnout and negative affect, and lower levels of life satisfaction and positive affect compared to non-YACs

No significant differences were observed in the levels of exhaustion, emotional impairment, cognitive impairment, negative and positive affect, and life satisfaction between YACs and non-YACs (all *p* > 0.05, see Table 3). The mean scores on the burnout sub-scales ranged from 2.9 to 3.2, suggesting that both YACs and non-YACs experience high levels of exhaustion, emotional impairment, and cognitive impairment [50]. The average sum score on the satisfaction with life scale are 21.6 and 22.1 for YACs and non-YACs, respectively, indicating that both YACs and non-YACs experienced slight life satisfaction [19]. Moreover, most of the YACs and non-YACs experienced reduced mental well-being (YACs: 77%, non-YACs: 72.4%) and lower life balance (YACs: 72%, non-YACs: 64%) in the times of COVID-19 than before.

### Relation between perceived life balance and psychological functioning

#### Hypothesis 4: Perceived life balance is negatively related to burnout (exhaustion, emotional impairment, and cognitive impairment) and negative affect and positively related to positive affect and life satisfaction in YACs and non-YACs

Perceived life balance is significantly inversely correlated with the exhaustion, emotional impairment, cognitive impairment, and negative affect, and positively correlated with positive affect and life satisfaction (all *p* < 0.001, see Table 4). This indicates that perceived life balance is negatively related to burnout and negative affect in both YACs and non-YACs, although the magnitude of the correlation of perceived life balance with cognitive impairment and negative affect is moderate in both YACs and non-YACs and weak for emotional impairment in non-YACs [51]. Perceived life balance is positively related to positive affect and life satisfaction in YACs and non-YACs, with a moderate correlation.

**Table 4:** Correlations between overall perceived life balance and psychological functioning

*Variables*	*YACs*	*Non-YACs*
	***Pearson coefficient with overall perceived life balance***	
Burnout		
Exhaustion	-.58*	-.54*
Emotional impairment	-.52*	-.33*
Cognitive impairment	-.55*	-.41*
Negative and positive affect		
Negative affect	-.55*	-.47*
Positive affect	.42*	.50*
Life satisfaction	.52*	-.50*

^*^Results are significant at a* p-*value of 0.05

## Discussion

As expected, the results from our study indicate that YACs perceive a lower life balance than non-YACs, albeit this difference is small. However, the two groups did not differ in their satisfaction with the time spent in most life areas (i.e., employment, education, and personal time), except for social life. That is, YACs were more satisfied with the time spent on social life compared to non-YACs. They also did not differ in the hours spent in different life areas, except for personal time, with YACs spending on average, 3.5 h per week less on personal activities than non-YACs. In addition, both groups did not differ with respect to their levels of burnout, negative and positive affect, and life satisfaction. In line with our hypothesis, both groups’ life balance was found to be inversely related to burnout and negative affect, and positively related to positive affect and life satisfaction.

Although YACs and non-YACs were equally satisfied with the time they spent in most of the life areas, YACs perceived overall slightly less balance in life than non-YACs. YACs experiencing less life balance as compared to non-YACs might be explained by our findings that YACs spent slightly less time on personal activities and, on average, an additional eight hours on caregiving, increasing their total time spent on life activities as compared to non-YACs. Thus, spending less time on personal activities and additional hours on caregiving might have resulted in an overall lower life balance among YACs when compared to non-YACs. However, this difference is small, which could be linked to the literature on ICGs that suggests that ICGs re-evaluate their life priorities as a results of caregiving. They are better able to adopt a positive mindset in facing adversity and set limits to balance their caregiving responsibilities and their own life [37, 49]. As most of the YACs in our study were providing care for more than three years, they may have already adjusted their lives and set goals that fit well with their caregiving role. For example, some YACs may have chosen educational and career paths that offer flexibility, such as educational programs with online courses or jobs in which they can work remotely, allowing them to better manage their caregiving duties [12]. They often seek part-time jobs with understanding employers who provide flexible work arrangements to manage employment with caregiving [15]. Therefore, it might be the case that YACs have succeeded in creating balance in activities between different life areas, resulting in only slightly less overall perceived balance than non-YACs. Another possible explanation for these findings could be that YACs may derive positive rewards from caregiving such as finding meaning in life and developing resilience through caregiving [18, 33, 43]. In the process of providing care, YACs may develop a close relationship with the care recipient, who is in most cases their grandparent or parent, and learn life lessons from them [18]. They may also develop a feeling of gratification by experiencing caregiving as an opportunity to give back to someone who has cared for them in the past [39]. Thus, allowing them to feel satisfied in most life areas, irrespective of spending slightly less time on personal activities.

Moreover, our results also show that YACs were more satisfied with the time spent on social life (including family and friends) compared to non-YACs, but did not significantly differ in the number of hours spent on social life. These findings could be linked to the caregiving status of YACs, where in line with the literature, our results suggest that majority of the YACs (see Table 2) provide occasional support to their care recipient [34]. Their caregiving role may be to support the primary ICGs who carry the main responsibility to support their care recipient. This implies that YACs may be spending their social time in a constructive and meaningful way by helping the primary ICG and their care recipient, thereby strengthening ties with family and friends. Thus, making YACs feel satisfied with the time they spend on social life.

Interestingly, there was no difference between YACs and non-YACs in experiencing burnout, negative and positive affect, and life satisfaction. Both groups experienced high levels of burnout and were slightly satisfied in life. The results are inconsistent with previous research reporting that YACs experience high negative psychological functioning (e.g., depression) and less positive psychological functioning (e.g., life satisfaction) as compared to non-YACs [10, 28, 30]. To provide a possible explanation for these findings, it will be useful to highlight the critical development phase of these young adults. Young adults, also known as ‘emerging adults,' are in a phase where they not only explore their identity in terms of their interests and the kind of life they would want, but also experience instability with respect to different life areas, in particular career and romantic relationships [6]. This phase can be exciting but is often daunting and confusing for young adults, who find themselves instable regarding their life decisions with respect to, for example, career or relationships. Moreover, in this phase, young adults move towards living an independent life and move out of their parent's house, which also implies that they are on their own having less support from their family. Literature suggests that more than half of young adults often experience anxiety, and a third report often feeling depressed in this phase of life [4]. Thus, this experience of lower mental well-being among young adults, including findings from our study where both YACs and non-YACs experience high burnout and only slight life satisfaction, might be due to a lack of clarity and instability regarding important life areas such as career and relationships.

In addition, it also needs to be noted that we have inconsistent findings with the literature regarding the comparison of psychological functioning between YACs and non-YACs, which could be due to the fact that our study was conducted during the COVID-19 pandemic. Most of the participants, both YACs and non-YACs reported that they experienced reduced mental well-being during COVID-19. In line with our results, previous research suggests that the COVID-19 pandemic may negatively impact student young adult's mental health, leading to higher levels of anxiety and depression [14, 57]. In most parts of the world, including the Netherlands, education went from classroom to online teaching [11, 44], also limiting opportunities for in-person social interactions.. Considering socialization is a crucial aspect of young adulthood, and the lockdown measures limited opportunities for in-person social interactions, COVID-19 may have had an impact on their psychological well-being, leading to high levels of burnout in both the groups. There is still a lack of clear understanding of the impact of caregiving on the psychological functioning of YACs, in particular for burnout and negative and positive affect, thus, more research needs to be done, for example, by replicating this study post-COVID-19 pandemic.

### Limitation and strengths

It is important to highlight certain limitations that could have hampered the interpretation of the results. The participants were predominantly female in both YACs (85.1%) and non-YACs (76.0%) group. This gender imbalance in our study sample may have influenced our finding as literature suggests that female ICGs are more likely to experience more caregiving burden and issues with mental health than male ICGs [2, 21, 53]. One of the reasons that we had more females in this study could be linked to the inclusion criterion of being a student. In the Netherlands, more young adult females are enrolled in college than males [20], potentially leading to recruitment bias in our study. We would also like to highlight that most often the caregiving role is undertaken by women as compared to men [47], which may have also added to gender imbalance in our YACs sample. We would also like to highlight on other recruitment biases this study may reflect. Firstly, we restricted our study to young adults who study in the Netherlands. Hence, these results may not apply to working YACs or non-YACs who are no longer following education or YACs who had to drop out of college due to their care responsibilities, making our sample not representative of the entire young adults population. However, we attempted to include participants from various sources and reasonable sample size to reduce the selection bias. It would be interesting to investigate in the future whether these results remain valid for YACs and non-YACs without student status. Secondly, in this study, we labeled young adults as caregivers if they take care of a loved one who is living with a disability, chronic illness, mental illness, old age problems, or substance abuse. In our definition, providing care may refer to a variety of caregiving tasks, including household tasks (e.g., cooking), personal care tasks (e.g., helping to dress), or emotional support (e.g., motivating their loved ones). However, literature on young caregivers suggests that growing up with a loved one having any illness may have a negative impact on young caregivers’ mental health, even if they do not provide ‘regular’, ‘substantial’ or ‘significant’ support to their loved ones [3]. Based on our definition, we may have missed YACs who are not actively involved in providing care to their ill loved ones, but who may experience a negative impact on their life balance and psychological functioning. Future research on YACs should be inclusive of young adults who are not performing caregiving tasks, but are living with their ill loved one, to explore the negative impact of the caregiving situation on YACs, irrespective of whether they provide caregiving tasks or not.

There were some methodological limitations as well. First, all participants completed the questionnaires in the same order. Although the survey took only 10–15 min to complete, the willingness to respond decreased somewhat over the course of completing the survey. This may have led to more missing values for approximately 10% of the participants who did not complete the questionnaires that were presented later. Moreover, our study data are cross-sectional rather than longitudinal; thus, causal conclusions cannot be drawn. Results from longitudinal data would be useful to establish a more comprehensive picture of the impact of caregiving on the YAC’s perceived life balance and psychological functioning over time when comparing YACs and non-YACs.

It is noteworthy to highlight some strengths of this study. To the best of our knowledge, this is the first quantitative study to use a control group of non-YACs to examine perceived life balance, burnout, and affect in YACs. Thus, it provides valuable insights into the impact of caregiving on perceived life balance and psychological functioning among YACs population to the limited yet growing literature on YACs. Moreover, the literature suggests that many YACs do not always recognise themselves as informal caregivers [34]. Therefore, in the survey, we did not explicitly ask participants about their caregiving status, but instead gave a broader definition of informal caregiving. Our definition could have helped YACs to recognise and choose their caregiving status better.

### Conclusion and future implication

This study indicated that YACs overall perceived slightly less balance in life than non-YACs. Although YACs and non-YACs did not differ in their satisfaction with time spent in most of the life areas, YACs were more satisfied with the time spent on social activities. They spent less hours on personal activities in comparison to non-YACs. However, YACs and non-YACs experienced similar levels of burnout, negative and positive affect, and life satisfaction. Both the groups experienced high levels of burnout and were only slightly satisfied in life. Thus, supporting young adults (both YACs and non-YACs) is important in improving their mental well-being and having a balanced life, especially during COVID-19. Healthcare professionals and school counselors, firstly need to recognise and create awareness among YACs regarding their role as a caregiver through, for example, joint awareness programs with the government. In addition, healthcare professionals and school counselors need to recognise the critical phase of young adults and provide the support that could, for example, help them reduce burnout and enhance their quality of life.

## Data Availability

The datasets used and/or analysed during the current study are available from the corresponding author on reasonable request.
